# A Cyan Fluorescent Reporter Expressed from the Chloroplast Genome of *Marchantia polymorpha*

**DOI:** 10.1093/pcp/pcv160

**Published:** 2015-12-03

**Authors:** Christian R. Boehm, Minoru Ueda, Yoshiki Nishimura, Toshiharu Shikanai, Jim Haseloff

**Affiliations:** ^1^Department of Plant Sciences, University of Cambridge, Downing Street, Cambridge CB2 3EA, UK; ^2^Department of Botany, Graduate School of Science, Kyoto University, Sakyo-ku, Kyoto, 606-8502 Japan; ^3^CREST, Japan Science and Technology Agency, Chiyoda-ku, Tokyo, 102-0076 Japan; ^4^Present address: RIKEN Center for Sustainable Resource Science, 1-7-22 Suehiro, Tsurumi, Yokohama, 230-0045 Japan.

**Keywords:** Chloroplast, Codon bias, GFP, *Marchantia polymorpha*, Reporter gene, Stromules

## Abstract

Recently, the liverwort *Marchantia polymorpha* has received increasing attention as a basal plant model for multicellular studies. Its ease of handling, well-characterized plastome and proven protocols for biolistic plastid transformation qualify *M. polymorpha* as an attractive platform to study the evolution of chloroplasts during the transition from water to land. In addition, chloroplasts of *M. polymorpha* provide a convenient test-bed for the characterization of genetic elements involved in plastid gene expression due to the absence of mechanisms for RNA editing. While reporter genes have proven valuable to the qualitative and quantitative study of gene expression in chloroplasts, expression of green fluorescent protein (GFP) in chloroplasts of *M. polymorpha* has proven problematic. We report the design of a codon-optimized *gfp* varian, *mturq2cp*, which allowed successful expression of a cyan fluorescent protein under control of the tobacco *psbA* promoter from the chloroplast genome of *M. polymorpha.* We demonstrate the utility of *mturq2cp* in (i) early screening for transplastomic events following biolistic transformation of *M. polymorpha* spores; (ii) visualization of stromules as elements of plastid structure in *Marchantia*; and (iii) quantitative microscopy for the analysis of promoter activity.

The nucleotide sequence of pCS CL0*b reported in this paper has been submitted to GenBank with accession number KT364744.

## Introduction

Reporter genes have enabled great contributions to our understanding of gene expression in a variety of organisms. Reporter genes that have been adapted for application in chloroplasts of algae and angiosperms include β-glucoronidase (Blowers et al. 1990, Staub and Maliga 1993), luciferase (Minko et al. 1999, Mayfield and Schultz 2003) and green fluorescent protein (GFP; Sidorov et al. 1999, Reed et al. 2001, Newell et al. 2003) alongside derivatives (Buhot et al. 2006). Reporter genes of the GFP family have become particularly popular as they are non-toxic (Millwood et al. 2008), do not require application of exogenous substrates, allow easy detection in vivo at high spatial resolution and have been shown to be expressed to high levels in chloroplasts, especially of seed plants (Sidorov et al. 1999, Reed et al. 2001, Newell et al. 2003). For these reasons, *gfp* is routinely used to test protocols for chloroplast transformation.

Recently, the liverwort *Marchantia polymorpha* has become increasingly popular as a basal plant model. Descending from the earliest terrestrial plants (Edwards et al. 1995, Wellman et al. 2003), *M. polymorpha* is a system well suited for the study of evolutionary effects associated with the transition from water to land. One notable characteristic of this lineage is the absence of mechanisms for RNA editing in chloroplasts of *M. polymorpha* (Ohyama et al. 2009). This is an exception among land plants (Malek et al. 1996, Freyer et al. 1997). The investigation of chloroplast-based gene expression in *M. polymorpha* benefits from its well-characterized chloroplast genome. The chloroplast genome of *Marchantia* was the first to be entirely sequenced (Ohyama et al. 1986), and has served as an important point of reference for >100 plastid genomes to follow (Cui et al. 2006). Furthermore, *M. polymorpha* is one of only a handful of plant species for which stable chloroplast transformation has been confirmed to date (Bock 2015). Three studies have reported generation of stable transplastomic lines of *M. polymorpha* by particle bombardment of 5- to 7-day-old sporelings, followed by propagation under selective conditions for several months to establish homoplasmy (Chiyoda et al. 2007, Ueda et al. 2012, Ueda et al. 2014). However, all three studies were limited to employing an *aadA* expression cassette conferring resistance to the antibiotic spectinomycin as the sole marker (Svab et al. 1990). As continuous strong expression of *aadA* is necessary for selection of transplastomic events and subsequent establishment of homoplasmy, this marker gene is inapplicable for monitoring and quantification of chloroplast-based gene expression. A fluorescent protein gene would provide a more suitable reporter. However, there are no previous reports of successful expression of a fluorescent reporter gene from the chloroplast genome of *M. polymorpha.*

Here, we report the design of an optimized *gfp* variant *mturq2cp*, which encodes a cyan fluorescent protein (CFP) successfully expressed from the *M. polymorpha* chloroplast genome. We introduce *mturq2cp* as a useful marker in early screening for transplastomic events following particle bombardment of *M. polymorpha* sporelings. We furthermore utilize *mturq2cp* to visualize stromules in *M. polymorpha*, and demonstrate the utility of *mturq2cp* as a reporter gene in quantitative gene expression studies.

## Results

As several studies have underlined the importance of modifying the codon usage of reporter genes to match target chloroplast DNA (cpDNA) both in seed plants (Reed et al. 2001) and in algae (Franklin et al. 2002), we chose to synthesize de novo a codon-optimized variant of the *gfp* reporter gene for expression in the *M. polymorpha* chloroplast genome. From the broad palette of *gfp* variants available, we chose the amino acid sequence of mTurquoise2 as the basis for synthesis of our codon-optimized fluorescent reporter for application in chloroplasts of *M. polymorpha.* mTurquoise2 has been reported to exhibit a high quantum yield (93%) compared with other fluorescent proteins (Goedhart et al. 2012), and expands the spectral palette of chloroplast-encoded fluorescent reporters to blue-green emission (λ_em_ = 474 nm).

**Fig. 1:**
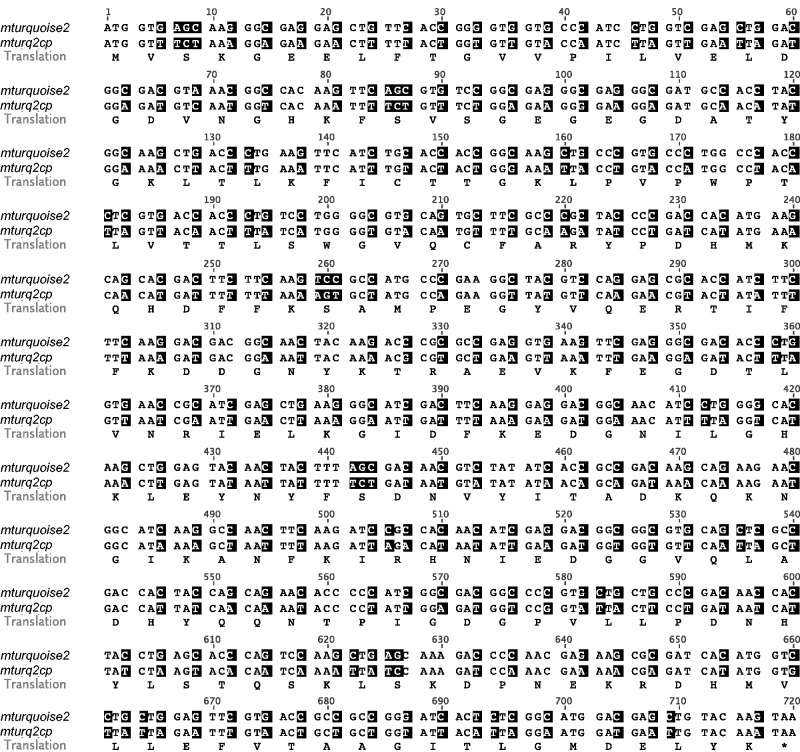
Comparison of the *mturquoise2* and *mturq2cp* coding regions. The amino acid sequence is shown below the aligned nucleotide sequences. Changed nucleotides are shaded.

The native nucleotide sequence of *mturquoise2* was modified to reflect the codon usage in liverwort chloroplasts (Kohchi et al. 1988; GenBank accession No. X04465). Codon optimization was guided by a commercial algorithm (Welch et al. 2009) based on codon usage information across 94 CDS (coding DNA sequences) encoded by the plastome of *M. polymorpha* (Nakamura et al. 1999). The GC3 value (defined as the average frequency of guanine or cytosine in the third codon position) of 12.50% in the codon-optimized gene which we call *mturq2cp* is markedly reduced compared with the native GC3 value of 96.25% in *mturquoise2*, and closely resembles the GC3 value of 12.10% in *M. polymorpha* chloroplast CDS (Fig. 1).

**Fig. 2:**
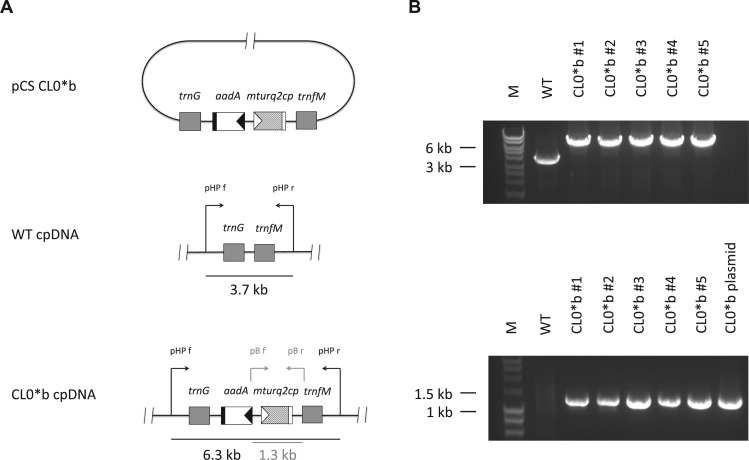
Generation and verification of homoplasmic chloroplast transformants. (A) Maps of the pCS CL0*b transformation vector (top), target region in the wild type (WT) cpDNA (middle) and the same region after integration of the cassette embracing the *aadA* and *mturq2cp* genes (bottom). Filled triangles indicate the rRNA operon promoter (black) and the *psbA* promoter (white) of the tobacco chloroplast genome, respectively. Filled rectangles indicate the *psbA* terminator of the tobacco chloroplast genome (black) and a hybrid 3′-untranslated region composed of the prokaryotic double terminator BBa_B0015 and the *rps16* terminator of the tobacco chloroplast genome (white), respectively. Black arrows indicate the position and orientation of the PCR primers pHP f and pHP r used for the detection of WT or transplastomic (CL0*b) cpDNA. Gray arrows indicate the position and orientation of the PCR primers pB f and pB r used for confirmation of the integrity of the reporter gene in transplastomic (CL0*b) lines. Primer sequences are provided in the Materials and Methods. (B) PCR analysis of genomic DNA isolated from WT and transplastomic (CL0*b) plants. Homoplasmy (top) and integrity of the reporter gene (bottom) were confirmed for transplastomic (CL0*b) lines after 4 months of repetitive subculture under selective conditions.

To test expression of the synthetic reporter gene *mturq2cp* encoded in the *M. polymorpha* chloroplast genome, a reporter plasmid pCS CL0*b was constructed (Fig. 2A). This vector was designed to integrate the *mturq2cp* reporter gene under control of the light-induced tobacco *psbA* promoter (Hayashi et al. 2003) alongside an *aadA* resistance cassette into the *trnG–trnfM* intergenic region of the *M. polymorpha* cpDNA by means of homologous recombination following microprojectile bombardment. After approximately 6 weeks of incubation under selective conditions, regenerating thalli were checked for fluorescence. 5 out of 12 spectinomycin-resistant plantlets obtained from the experiment exhibited punctate cyan fluorescence, and as candidate plastid transformants were subjected to repetitive subculture for establishment of homoplasmy. Homoplasmy and integrity of the inserted reporter gene were confirmed for all candidate lines 4 months after microprojectile bombardment by PCR analysis of the transformed plastid DNAs (Fig. 2B).

**Fig. 3:**
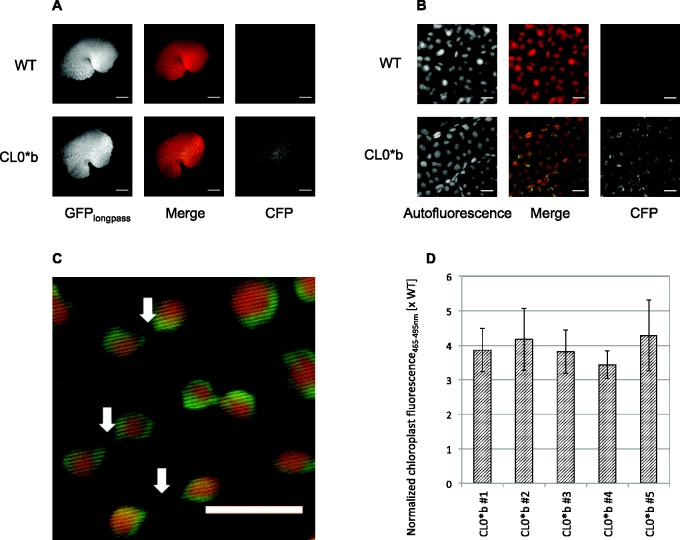
Analysis of chloroplast fluorescence in vivo. (A) Widefield micrographs of wild type (WT) and transplastomic (CL0*b) thalli imaged under GFP_longpass_ and CFP filter settings. Scale bar = 1 mm. (B) Confocal micrographs of WT and transplastomic (CL0*b) thalli imaged under Autofluorescence and CFP channel emission settings. Scale bar = 10 µm. (C) Stromules visualized by chloroplast-localized mTurquoise2 in transplastomic (CL0*b) *M. polymorpha* (white arrows). Shown is a subsection of image CL0*b Merge from (B). Scale bar = 10 µm. (D) Normalized chloroplast fluorescence_465–495 nm_ of transplastomic (CL0*b) lines relative to the WT. Three different thallus sections from each line were subjected to confocal imaging to capture micrographs under Autofluorescence and CFP channel emission settings upon excitation using the argon laser at 458 nm. Chloroplast-localized CFP channel intensity was normalized for tissue depth via the corresponding Autofluorescence channel intensity. Error bars represent the SD of normalized chloroplast fluorescence over the cyan spectral window 465–495 nm between three different thallus sections.

While punctate chloroplast-localized cyan fluorescence was evident under a low-magnification widefield microscope used for screening (Fig. 3A), high-resolution confocal microscopy was employed to specify the localization of the signal to the chloroplast stroma (Fig. 3B). The brightness of cyan fluorescence in homoplasmic transformants allowed the visualization of fine tubules extending from individual plastids in *M. polymorpha*, which are believed to be stromules (Senn 1908, Köhler et al. 1997) (Fig. 3C). To demonstrate the utility of *mturq2cp* for analysis of chloroplast promoter activity in vivo, confocal micrographs of thalli from wild-type and transplastomic plants were subjected to image processing: binary masks of plastid positions were created based on confocal Autofluorescence channel (emission 610–700 nm) micrographs, and normalized chloroplast fluorescence_465–495 nm_ calculated by dividing the average CFP channel (emission 465–495 nm) pixel value across each plastid particle by the corresponding average Autofluorescence channel pixel value to account for differences in plastid tissue depth. Taking this approach, we found the normalized chloroplast fluorescence_465–495 nm_ in homoplasmic CL0*b transformants to exceed the background in the wild type by approximately 4-fold (Fig. 3D). Normalized chloroplast fluorescence_465–495 nm_ was consistent across all independent transplastomic lines.

**Fig. 4:**
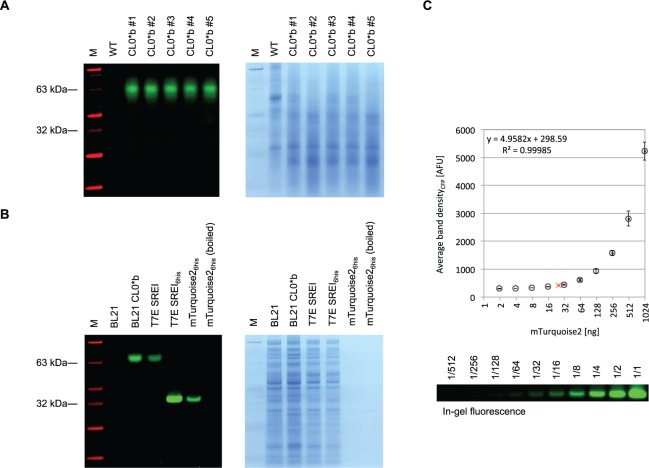
Detection of chloroplast-expressed mTurquoise2 in transgenic lines of *Marchantia polymorpha.* (A) *M. polymorpha* protein extracts separated by SDS–PAGE. Protein extracts from wild type (WT) and transplastomic (CL0*b) lines of *M. polymorpha* were separated by 4–12% SDS–PAGE, and visualized by in-gel fluorescence (left) and subsequent Coomassie stain (right). The fluorescence image was generated using a custom imaging device described in the Materials and Methods for visualization of CFP bands (emission 486/10 nm, green) and marker (emission 540/10 nm, red). (B) Untagged and His_6_-tagged mTurquoise2 separated by SDS–PAGE. Untagged mTurquoise2 was expressed from plasmids pCS CL0*b (*psbA* promoter) in BL21 *E. coli* and pCRB SREI (T7 promoter) in T7 Express *E. coli.* His_6_-tagged mTurquoise2 was expressed from plasmid pCRB SREI_6his_ (T7 promoter) and purified by affinity chromatography. The rightmost lanes contain 50 ng of unboiled and boiled purified protein, respectively. Visualization by in-gel fluorescence and Coomassie stain was conducted as described above. (C) Standard curve for quantification of mTurquoise2 based on in-gel fluorescence. Serial dilutions of 1 µg purified of mTurquoise2 were separated by 4–12% SDS–PAGE and the band intensity quantified by in-gel fluorescence as described in the Materials and Methods. Levels of mTurquoise2 extracted from transplastomic (CL0*b) *M. polymorpha* (red cross) were estimated by linear regression analysis on densities of fluorescent target bands as shown in (A). Error bars represent the SD of average band density between three different experiments.

To confirm that the significant increase in cyan fluorescence localized in the stroma of *M. polymorpha* chloroplasts transformed by the pCS CL0*b vector is a result of the expression of mTurquoise2, plant protein extracts were subjected to SDS–PAGE followed by detection of in-gel fluorescence. In contrast to extracts from wild type *M. polymorpha*, extracts from CL0*b chloroplast transformants contained a protein band detectable by in-gel fluorescence near the 63 kDa marker band (Fig. 4A). As suggested by identically processed controls consisting of untagged and His_6_-tagged mTurquoise2 expressed in *Escherichia coli*, this apparent size does not indicate the presence of a protein dimer but is a consequence of retarded migration during PAGE conducted under partially denaturing conditions (Fig. 4B). Based on a standard curve (Fig. 4C), the concentration of mTurquoise2 in transplastomic *M. polymorpha* was estimated at approximately 20 ng mTurquoise2 per mg of thallus fresh weight.

## Discussion

GFP is the most widely utilized marker in the biological sciences, and has greatly contributed to our understanding of gene expression, protein localization and interaction, and dynamic cellular events (Zimmer 2002, Chudakov et al. 2010). However, several modifications to the original *gfp* gene from *Aequorea victoria* were necessary to allow sufficient expression and brightness for wide application as a marker in plant systems: these include removal of a cryptic intron (Haseloff et al. 1997), improvement of protein solubility (Davis and Vierstra 1998), brightness (Pang et al. 1996) and optimization of codon usage (Chiu et al. 1996) of the *gfp* gene to match the nuclear genome of the target organism. While early application of GFP in chloroplasts involved targeting of the nuclear-expressed gene product to chloroplasts using a transit peptide (Köhler et al. 1997), the *gfp* gene soon after proved its utility as a transformation marker expressed in chloroplasts of several seed plants, including tobacco (Khan and Maliga 1999, Reed et al. 2001), potato (Sidorov et al. 1999), lettuce (Lelivelt et al. 2005), sugar beet (De Marchis et al. 2009) and alfalfa (Wei et al. 2011).

The liverwort *M. polymorpha* has recently attracted attention as a basal multicellular plant model due to its morphological and genomic simplicity (Yamato and Kohchi 2012), rapid life cycle (Shimamura 2012) and simplicity of nuclear transformation (Ishizaki et al. 2008). Known as the source of the first plastid DNA genome sequence to be reported (Ohyama et al. 1986), the chloroplast of *Marchantia* is an interesting platform for plastid gene expression studies due to its relationship to the earliest terrestrial plants (Edwards et al. 1995, Wellman et al. 2003). Aspects of evolutionary divergence between liverwort and angiosperm chloroplasts have been investigated in the context of RNA editing (Ohyama et al. 2009), photosynthesis-related enzymes (Ueda et al. 2012, Ueda et al. 2014) and sigma factors (Ueda et al. 2013). For a decade after the initial demonstration of transformation of chloroplasts in *M. polymorpha* by particle bombardment (Chiyoda et al. 2007), the *aadA* resistance marker remained the only heterologous gene to be successfully expressed in this organelle. Despite the utility of *gfp* and its variants as reporter genes, there are no previous reports of their successful expression from the chloroplast genome of *M. polymorpha.* We chose to synthesize an optimized *gfp* variant de novo based on the codon usage of *M. polymorpha* cpDNA (Kohchi et al. 1988; GenBank accession No. X04465). This approach was encouraged by previous reports of codon optimization markedly increasing the expression of *gfp* in chloroplasts of the seed plant tobacco (Reed et al. 2001) and the unicellular alga *Chlamydomonas reinhardtii* (Franklin et al. 2002). We chose to optimize *mturquoise2* which encodes the fluorescent protein exhibiting the highest reported quantum yield (93%; Goedhart et al. 2012) to facilitate in vivo detection. While CFP has been previously targeted to and detected in plastids (Nelson et al. 2007), expression from a plant chloroplast genome has, to best of our knowledge, not been reported before. The emission spectrum of *mturq2cp* (λ_em_ = 474 nm) introduced herein complements proven green- (λ_em_ = 507 nm; Sidorov et al. 1999, Reed et al. 2001, Lelivelt et al. 2005) and yellow- (λ_em_ = 527 nm; Buhot et al. 2006) emitting plastid-encoded reporters.

In the process of adjusting the *mturquoise2* gene to codon usage in *M. polymorpha* chloroplasts, 212 out of 240 codons were synonymously changed. Codon optimization was evenly distributed across the gene, with 72, 71 and 69 codons changed over the first, second and third segments of 80 codons, respectively. The original *mturquoise2* sequence includes 16 (6.67%) optimal (the triplet in question is most frequently used among synonyms in *M. polymorpha* chloroplast CDS) and 160 (66.67%) rare (the triplet in question has a frequency of ≤10% among synonyms in *M. polymorpha* chloroplast CDS) triplets, and the optimized *mturq2cp* gene is composed of 174 (72.5%) optimal and 15 (6.25%) rare triplets. After codon optimization, the native GC3 value of 96.25% in *mturquoise2* was reduced to 12.50% in the codon-optimized gene *mturq2cp*, closely resembling the GC3 value of 12.10% in *M. polymorpha* chloroplast CDS. The mean codon frequency difference between reporter gene codon usage and *M. polymorpha* chloroplast codon usage was 4.59%. The *mturq2cp* gene is thus significantly better adapted for efficient expression in chloroplasts of *M. polymorpha* than non-codon-optimized *mturquoise2* (65.72%).

Introduction of the *mturq2cp* expression cassette into *M. polymorpha* chloroplasts was mediated by homologous recombination. We chose to target the *trnG–trnfM* intergenic spacer (large single copy region of cpDNA; Ruf et al. 2001) instead of the *trnI–trnA* intergenic spacer (inverted repeat region of cpDNA) initially utilized for transformation of *M. polymorpha* chloroplasts (Chiyoda et al. 2007) to prevent gene conversion-mediated transgene loss in a heteroplasmic population (Lutz et al. 2007). The transformation efficiency (defined as the percentage of regenerating thalli containing the transgene) of 41.7% observed in the course this study was comparable with that in the reference report (66.7%; Chiyoda et al. 2007). Antibiotic-resistant plantlets could be identified with high confidence on selection plates (approximately 4–6 weeks post-bombardment). Positive chloroplast transformants were easily distinguished based on their fluorescence under a widefield fluorescence microscope equipped with a suitable GFP or CFP emission filter (Fig. 3A). This underlines the utility of constitutively expressed *mturq2cp* as a quick, simple and non-invasive chloroplast transformation marker in *M. polymorpha.* The availability of a second transformation marker functional in chloroplasts of *M. polymorpha* alongside the *aadA* cassette is particularly useful in the case of high rates of false positives after spectinomycin selection with moderate to high cell densities (Chiyoda et al. 2007).

Beyond simple screening for transgenic events, the brightness of mTurquoise2 in homoplasmic transformants allowed visualization of stromules (Köhler et al. 1997) connecting individual chloroplasts in *M. polymorpha* (Fig. 3C). This result substantiates initial observations of a peristromium in *Marchantia* dating back to the early 20th century (Senn 1908).

We have furthermore demonstrated that chloroplast-localized fluorescence resulting from expression of *mturq2cp* can be easily quantified by means of confocal microscopy and common image analysis techniques (Fig. 3D). Expression of the reporter driven by the tobacco *psbA* promoter could be detected by normalized chloroplast fluorescence_465–495 nm_, which significantly exceeded background level and was consistent across independent lines. The synthetic gene will therefore be useful in future gene expression studies in *M. polymorpha* chloroplasts.

Electrophoresis of extracts from transplastomic *M. polymorpha* under semi-denaturing conditions showed in-gel CFP fluorescence, with an apparent size of approximately 63 kDa (Fig. 4). This is lower than the expected mobility of mTurquoise2 (mol. wt. of 27 kDa). We reasoned that the observed slow mobility of unboiled samples during SDS–PAGE was not indicative of the formation of protein dimers. The CFP mTurquoise2 is an obligate monomer, containing the mutations Y66W, N146I and A206K (von Stetten et al. 2012). Unboiled GFP protein in the presence of SDS is capable of forming partially denatured conformations which retain fluorescence, and these extended conformations possess slower mobility under conditions of PAGE (BioRad Laboratories Inc. Application Note #M1660023). Incomplete denaturation and consequent low mobility is consistent with our observations of in-gel fluorescence for unboiled mTurquoise2 during SDS–PAGE. Samples of untagged and His_6_-tagged mTurquoise2 expressed in *E. coli* were also analyzed by SDS–PAGE. The mobility and in-gel fluorescence properties of the bacterial untagged mTurquoise2 samples were similar to those of the plant extracts. The addition of a His_6_-tag resulted in a marked increased in mobility of the partially denatured mTurquoise2 samples (Fig. 4B), perhaps due to differences in conformation or SDS binding, which have previously been reported as a major determinant in explaining anomalous protein migration behavior (Rath et al. 2009, Shi et al. 2012). The mobility of boiled samples was consistent with the expected size of the protein.

Successful expression of mTurquoise2 from the chloroplast genome facilitates the implementation of powerful two-color techniques in plastids. In this context, the blue-green-(λ_em_ = 474 nm) emitting fluorescent protein encoded by *mturq2cp* complements proven yellow-emitting chloroplast reporters (Buhot et al. 2006). One notable application is fluorescent protein FRET (fluorescence resonance energy transfer) which has enjoyed wide popularity as a method for the elucidation of dynamic protein interactions in vivo for many years (Piston and Kremers 2007). A variety of chloroplast-based dynamic protein complexes involved in photosynthesis and secondary metabolism are attractive targets for this technique, yet its application has been limited to chloroplast-targeted proteins to date (Seidel et al. 2010). Complemented by a proven yellow reporter gene, the bright cyan reporter encoded by *mturq2cp* expands the scope of plastid-based FRET to plastid-encoded as well as plastid-targeted proteins. Another two-color technique we strive to adopt in chloroplasts following this study is the ratiometric characterization of gene expression in planta (Federici et al. 2012). Taking into account environmental factors modifying overall gene expression capacity, the ratiometric approach is well suited for quantifying the activity of native, heterologous or synthetic promoters to be employed in future genetic engineering efforts on the chloroplast genome.

## Materials and Methods

### Plant materials and growth conditions

Male and female plants of *M. polymorpha* Cam-1 and Cam-2, respectively, were grown on half-strength Gamborg’s B5 medium containing 1.2% agar under continuous white light.

### Construction of plastid transformation vector

The codon-optimized *mturq2cp* gene (see GenBank accession No. KT364744) was synthesized by DNA2.0, its coding sequence linked to a double terminator (BBa_B0015) and the oligonucleotide amplified using primers 5′-GAGGAAAAAATGGTTTCTAAAGGAGAAGAACTTTTTACTG-3′ and 5′-ATTTCTCTAGAACTAGAAATGTATAAACGCAGAAAGGCCCAC-3′. The resulting fragment was inserted into the pCS GFP vector (GenBank accession No. LC068606) backbone amplified using primers 5′-GCGTTTATACATTTCTAGTTCTAGAGAAATTCAATTAAGGAA-3′ and 5′-GTTCTTCTCCTTTAGAAACCATTTTTTCCTCCGGATCCCC-3′, by means of isothermal assembly (Gibson et al. 2009). Prior to plastid transformation, the resulting vector was linearized using *Not*I.

### Plastid transformation and visual screening for transplastomic events

Chloroplasts of *M. polymorpha* were transformed by particle bombardment as previously described (Chiyoda et al. 2008), with minor modifications: 5-day-old sporelings were bombarded using a biolistic delivery system (Bio-Rad), and bombarded cells were incubated overnight under continuous light at 20°C. Bombarded cells were then divided onto four sucrose-free selective half-strength Gamborg’s B5 medium plates containing 500 mg l^–1^ spectinomycin. Repetitive subculture in the same growth conditions was employed to establish homoplasmic lines. Plate-based visual screening of antibiotic-resistant thalli for expression of the fluorescent reporter was conducted by means of a Leica MZ FLIII fluorescent stereomicroscope (Leica Microsystems) equipped with Leica GFP1 (excitation 425/60, emission 480 long pass) or CFP (excitation 436/20, emission 480/40) filter sets. Under the Leica GFP1 filter set, transformed chloroplasts expressing the *mturq2cp* gene were discernible by green-yellow signal distinct from the red autofluorescence exhibited by wild-type chloroplasts.

### PCR analysis

Approximately 10 mg of thalli were disrupted in 100 µl of DNA extraction buffer (100 mM Tris–HCl pH 9.5, 1 M KCl, 10 mM EDTA), and boiled at 98°C for 5 min. Following 2 min on ice and addition of 400 µl of sterile H_2_O, 1 µl of the resulting extract was used directly as a template for PCR. PCR was carried out using KOD Hot Start DNA Polymerase (Merck Millipore) following the manufacturer’s instructions under the following cycling conditions: 35 cycles of 94°C for 15 s, 56°C for 30 s and 68°C for 8 min. The primer pair pHP f (5′-GGTATTTACGATACATGGGCTC-3′) and pHP r (5′-TGGCTTACTGATATTGCTCACC-3′) annealing to *M. polymorpha* cpDNA outside of regions spanned by the *trnG* and *trnfM* homology arms was employed for confirmation of transplastomic integration events and homoplasmy. Primers pB f (5′-TTCAAATTCGCCCGGAG-3′) and pB r (5′-GAGCTCGGAATTCAATGGAAG-3′) annealing to the rRNA operon promoter and the GFP cassette terminator, respectively, were employed for confirmation of the integrity of the reporter gene.

### Quantification of chloroplast-localized fluorescence

High-resolution imaging of *M. polymorpha* chloroplasts within intact thallus segments was performed using a Leica SP5 Confocal Laser Scanning Microscope (Leica Microsystems) equipped with a HCX PL APO × 63 1.2 W objective. mTurquoise2 was excited using the argon ion laser at 458 nm, and CFP fluorescence was detected across the 465–495 nm spectral window. Autofluorescence of chloroplasts was detected across the 610–700 nm spectral window. 1,024 × 1,024 pixel images were captured at 100 Hz scanning speed and image averaging of three at a total magnification of×189. Raw confocal images were analyzed using the open source image processing package Fiji (Schindelin et al. 2012) as follows: the Autofluorescence channel of each image was used to create a mask of plastid positions by rolling ball background subtraction (*r* = 5 pixels), binary transformation and application of the Watershed algorithm. Normalized chloroplast fluorescence_465–495 nm_ was calculated by means of dividing the average CFP channel pixel value across each plastid particle by the corresponding average Autofluorescence channel pixel value to account for different tissue depths of chloroplasts.

### Preparation of purified mTurquoise2

Plasmid pCRB SREI for pT7-driven expression of mTurquoise2 was generated by amplification of the *mturquoise2* coding sequence using primers 5′-AGAGAAAGAGGAGAAATACTAGATGGTGAGCAAGGGCG-3′ and 5′-GCCTGGCTCTAGTATTATTACTTGTACAGCTCGTCCATG-3′, and insertion of the resulting fragment downstream of a T7 promoter (BBa_I712074) and ribosomal binding site (BBa_B0034) into a high copy number backbone (pSB1A3) amplified using primers 5′-GCTGTACAAGTAATAATACTAGAGCCAGGCATCAAATAA-3′ and 5′-CTCACCATCTAGTATTTCTCCTCTTTCTCTAGTATGCA-3′ by means of isothermal assembly (Gibson et al. 2009). Using pCRB SREI as a template, plasmid pCRB SREI_6his_ encoding mTurquoise2 fused to an N-terminal His_6_-tag was generated following a similar approach amplifying the *mturquoise2* coding sequence using primers 5′-ATACTAGATGGGTTCTTCTCACCATCACCATCACCATGGTTCTTCTGTGAGCAAGGGCGAGGAG-3′ and 5′-GCCTGGCTCTAGTATTATTACTTGTACAGCTCGTCCATG-3′, and the vector backbone using primers 5′-GCTGTACAAGTAATAATACTAGAGCCAGGCATCAAATAA-3′ and 5′-AACCATGGTGATGGTGATGGTGAGAAGAACCCATCTAGTATTTCTCCTCTTTCT-3′. Recombinant mTurquoise2 protein to be used as a positive control was expressed in T7 Express competent *E. coli* (New England Biolabs) transformed by pCRB SREI_6his,_ and purified under native conditions using an Ni-NTA Fast Start kit (Qiagen) according to the manufacturers’ instructions.

### Visualization of proteins by in-gel fluorescence and coomassie staining

For total protein extraction from *M. polymorpha* tissues, 50 mg (fresh weight) of thallus were ground by liquid nitrogen and the resulting powder vortexed in 200 µl of 2 × Tris–glycine for 30 s. For total protein extraction from BL21 or T7 Express competent *E. coli* (New England Biolabs), an aliquot of overnight culture containing approximately 0.5–1 × 10^9^ cells was pelleted by centrifugation at 14,000 r.p.m. and 4°C for 1 min, resuspended in 50 µl of 2 × Tris–glycine SDS sample buffer and vortexed for 30 s. In both cases, 2-fold dilutions of crude extracts were centrifuged at 14,000 r.p.m. and 4°C for 20 min to remove cell debris, and unboiled (unless indicated otherwise) supernatants were directly loaded onto a NuPAGE Novex 4–12% Bis–Tris protein minigel (Life Technologies). Sample proteins were separated by SDS–PAGE in MES SDS running buffer over 35 min at 200 V, and the gel subsequently was shaken in dH_2_O for 3 × 5 min to remove excess SDS and buffer salts. In-gel fluorescent visualization was carried out in a custom imaging device consisting of an optical breadboard and frame (Thorlabs) on which were mounted LED light sources (Royal-Blue 447.5 nm and Cyan 505 nm A Rebel Star CoolBase LEDs, Luxeon Star LEDs). The output was collimated with a lens (Carclo Optics), filtered with excitation short pass filters (Comar Optics) of 450 and 510 nm, respectively, and shaped by engineered top hat diffusers (ED1-C50-MD; Thorlabs). A monochromatic camera (CoolSNAP HQ2; Photometrics) with a zoom lens and 10 nm bandpass filters (Edmund OpticsA) of 486 and 540 nm, respectively, in a filter wheel was used to collect the emission over 10 s. Average band density was measured by means of the Mean Gray Value function implemented in the Fiji package (Schindelin et al. 2012). Following analysis of in-gel fluorescence, gels were stained by EZBlue Gel Staining Reagent (Sigma-Aldrich) according to the manufacturer’s protocol, and excess dye removed by shaking in dH_2_O overnight.

## Funding

This work was supported by the Gates Cambridge Trust [Gates Cambridge Scholarship to C.R.B.]; the Japan Society for the Promotion of Sciences [KAKENHI grant No. 25650126 to M.U., the Next Generation World-Leading Researchers grant No. GS015 from the Precursory Research for Embryonic Science and Technology to Y.N.]; the Biotechnology and Biological Sciences Research Council and Engineering and Physical Sciences Research Council [OpenPlant grant No. BB/L014130/1 to J.H.].

## Abbreviations

CFP: cyan fluorescent protein
CDS: coding DNA sequence
cpDNA: chloroplast DNA
FRET: fluorescence resonance energy transfer
GFP: green fluorescent protein
